# Non-Coding RNAs in Peritoneal Carcinomatosis: From Bench to Bedside

**DOI:** 10.3390/cancers16172961

**Published:** 2024-08-25

**Authors:** Julia Bohosova, Nida Sarosh Ashraf, Ondrej Slaby, George A. Calin

**Affiliations:** 1 Translational Molecular Pathology Department, The University of Texas MD Anderson Cancer Center, Houston, TX 77030, USA; julia.bohosova@ceitec.muni.cz (J.B.); sp20-pbm-002@isbstudent.comsats.edu.pk (N.S.A.); 2 Central European Institute of Technology, Masaryk University, 62500 Brno, Czech Republic; oslaby@med.muni.cz; 3 Department of Biology, Faculty of Medicine, Masaryk University, 62500 Brno, Czech Republic; 4 Cancer Genetics and Epigenetics Lab, Department of Biosciences, COMSATS University Islamabad, Islamabad 45550, Pakistan; 5 The RNA Interference and Non-Coding RNA Center, The University of Texas MD Anderson Cancer Center, Houston, TX 77030, USA

**Keywords:** peritoneal carcinomatosis, non-coding RNA, miRNA, ascites, exosomes

## Abstract

**Simple Summary:**

Peritoneal carcinomatosis is a term for cancer cells spreading from tumors of internal organs and massively invading a large part of the membrane lining the abdomen and pelvis. For most patients, peritoneal carcinomatosis suggests only several months of life left. Current medicine can offer only alleviation of symptoms from this incurable disease. Researchers are intensely exploring some new therapeutic targets. Among promising candidates are non-coding RNAs, short molecules serving as important regulators in cells. When a disease such as cancer develops in the body, it is accompanied by typical changes in levels of non-coding RNAs. In this review, we provide an overview of current state of knowledge regarding the changes of non-coding RNA levels in peritoneal carcinomatosis. Deeper understanding of this topic could lead to the identification of non-coding RNAs as feasible specific biomarkers or novel therapeutic targets in the treatment of peritoneal carcinomatosis.

**Abstract:**

Peritoneal carcinomatosis represents an advanced stage of tumors within the peritoneal cavity. Once considered an incurable terminal cancer metastasis, contemporary medicine is on the hunt for certain potentially curative options alongside the present day’s palliative disease management. However, for most patients, peritoneal carcinomatosis continues to pose a fatal late-stage prognosis with a grim future outlook. Over the past two decades, non-coding RNAs have garnered significant attention due to their undeniable significance in regulating cellular processes across all levels. Disruption of the intricate regulation led by non-coding RNAs has been demonstrated to have a substantial impact on various human diseases, particularly in cancer, including solid tumors originating from the organs of the peritoneal cavity. This review aims to offer a comprehensive overview of the current state of knowledge in the under-researched field of peritoneal carcinomatosis, focusing specifically on the role of non-coding RNAs in the development of this condition and delineating potential avenues for future research.

## 1. Why Study Non-Coding RNAs in Peritoneal Carcinomatosis?

The term peritoneal carcinomatosis (PC) was first coined in 1931 to describe an invasion of the peritoneal wall by ovarian cancer cells [1,2]. Up until today, peritoneal carcinomatosis, peritoneal carcinosis, or peritoneal dissemination usually referred to the state when solid cancer spreads to the peritoneal lining, or peritoneal wall [3]. The emphasis is on the word “usually,” as the use of the term peritoneal carcinomatosis varies. Some authors consider carcinomatosis as simply a metastatic invasion of the peritoneal wall by the tumors of internal organs [1,4,5]. In contrast, others differentiate between peritoneal metastases and PC, as the widespread dissemination and development of several tumors blanketing large areas of the organ or body part [1,5,6,7,8]. The most common primary tumor sites are the ovaries, the stomach, and the colon, followed by tumors of other intraperitoneal organs [9]. Although PC arising from cancer of extraperitoneal origin does exist, it is very rare, occurring in up to 10% of all peritoneal metastasis cases [1].

Reliable epidemiological data are still very limited and rely mostly on regional databases and cancer registries [10]. The PC incidence is inferred from the incidence of advanced primary malignancies that give rise to the peritoneal metastases and eventually also to PC. It is estimated that about 2 to 3/10,000 individuals per year have PC arising from colorectal cancer, while 1.6 per 1 million people/year will develop it from neuroendocrine tumors of the gastrointestinal tract [1,11,12,13]. Exact molecular mechanisms of PC etiology remain poorly understood [4], although some have been already outlined (reviewed in Cortes-Guiral et al. [6]).

Following the general lack of epidemiological data, there is no specific screening program for PC, or for peritoneal cancer, for that matter. However, regional screening programs focused on intraperitoneal cancer such as colorectal cancer and their effectiveness goes hand in hand also with the incidence of PC, as detection of cancer in lower stages leads to better treatment options and overall prognosis for patients [6].

The extensive spread of cancer throughout the peritoneal wall is synonymous with advanced disease. PC presents a significant clinical challenge, especially since intraperitoneal cancers are often diagnosed at an advanced stage. For instance, in gastric cancer (GC), the third most deadly cancer globally [14,15], PC can be detected synchronously with the initial diagnosis in 15–40% of patients, with a median survival of 4–6 months [15,16]. In the remaining cases, the peritoneum is a common site of recurrence [15,17]. Therefore, the presence of PC signifies a very poor prognosis. Novel therapeutic options such as cytoreductive surgery combined with hyperthermic intraperitoneal chemotherapy (HIPEC) as well as pressurized intraperitoneal aerosol chemotherapy (PIPAC) [4,6] enable longer overall survival of some PC patients [3,18,19]. However, the patients for this type of treatment need to be carefully selected [20,21]; thus, for most of the affected, a PC verdict means a fatal late-stage condition oftentimes managed using only palliative care approaches [5,22]. As Harada et al. concluded in their review specifically on GC, none of the modern treatment modalities that have been explored in peritoneal metastases exhibited remarkable effects and therefore have not yet become a standard treatment [4,20]. Moreover, targeted treatment has been revolutionized and greatly expanded in recent decades but lags significantly in the case of PC and peritoneum-related malignant diseases, suggesting that there is great potential not only for already available therapeutic options to be tested and administered but also for personalized therapy utilizing genetic and epigenetic markers [20].

In the last two decades, non-coding RNAs (ncRNAs) have gained significant research attention due to their undeniable importance in the regulation of cellular life on all levels. ncRNAs define a heterogeneous group of transcripts that are not translated into proteins but regulate protein expression and function through various mechanisms [23]. Disruption of the delicate ncRNA-led regulation has been shown to have a major impact on many human diseases, most dominantly in cancer, including solid tumors of intraperitoneal organs [24,25]. Even though there is already a long list of publications focused on the dysregulation and role of ncRNAs in peritoneal metastases, there have been just a handful addressing PC specifically. In this review, we would like to summarize and discuss the current state of knowledge in the under-researched area of PC and, more specifically, the role of non-coding RNAs in the development of this condition, and to outline possible new avenues of future research.

## 2. How Did We Select the Information?

**Figure 1:**
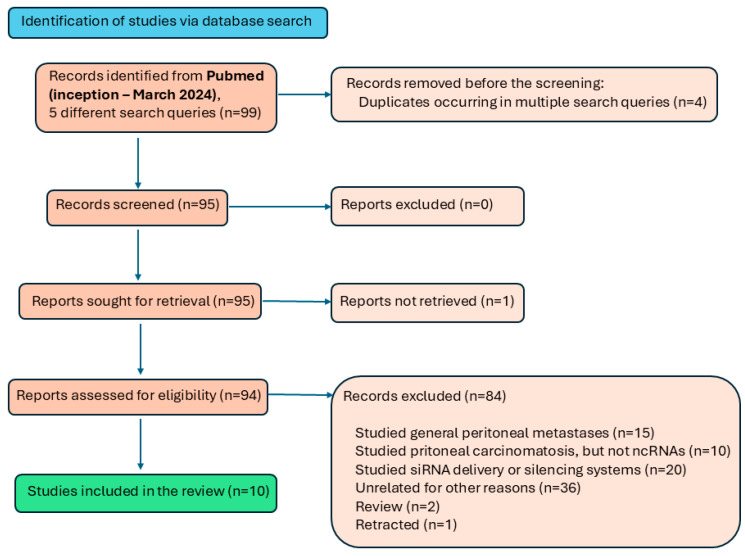
Literature selection flow chart.

For the search of relevant publications, we used the PubMed database and several different search queries to maximize the possibility of finding all relevant publications published by March 2024. All results were checked manually. Only original, unretracted research articles focused on the role or dysregulation of non-coding RNAs in peritoneal carcinomatosis or peritoneal dissemination were deemed relevant; articles focused on peritoneal metastases were excluded. The literature selection flow chart (Figure 1) illustrates the search process. 

The first search query (peritoneal gastric carcinomatosis non-coding RNA) returned 36 results, out of which we considered relevant one article. The second search query (miRNA peritoneal carcinomatosis) returned 159 results, three of which were relevant. The third search query (((peritoneal) AND (carcinomatosis)) NOT (carcinoma)) AND (miRNA)) identified eight results, four of which were relevant. The fourth search query (((peritoneal) AND (carcinomatosis)) NOT (carcinoma)) AND (ncRNA)) identified 22 results, five of which were relevant, partially overlapping with the second and the third search. The fifth search query (((peritoneal) AND (carcinomatosis)) NOT (carcinoma)) AND (lncRNA)) did not return any results. For this review article, we thus identified ten articles (overview in Table 1), which will be discussed in the following paragraphs.

## 3. What Did We Learn about Non-Coding RNAs in Peritoneal Carcinomatosis?

Although PC presents a unique yet largely unfulfilled medical need, the research on the role of ncRNAs in this fatal condition is waiting for its prime. Even though there is already a handful of articles published, the study design is diverse in the techniques and cohorts or samples analyzed, which also limits the comparability of the results. 

Nevertheless, current data outline the inevitable involvement of ncRNAs and their dysregulation also in PC, as in any other tumor type studied in recent decades. Building on the strong base of ncRNA research in localized as well as metastatic solid cancer, one of the first studies analyzed two well-known long non-coding RNAs (lncRNAs), MALAT1 and HOTAIR, in gastric cancer patients [26]. These two transcripts have been known to be associated with the development of solid cancer [36,37] with cancer progression and metastasis; however, a study by Okugawa et al. was the first evidence of the role of lncRNAs in PC. In their study, they analyzed a cohort of 150 Japanese patients and the paired tumor and non-tumor tissue. The effect of HOTAIR and MALAT was also tested on human GC cell lines and the effect of HOTAIR was studied in vivo. The results show that the expression of both lncRNAs was significantly higher in cancer tissue compared to the non-tumor tissue. Moreover, high expression of HOTAIR was associated with metastases and poor outcomes in GC patients. The team showed that HOTAIR suppresses cell proliferation, tumorigenicity, migration, and invasion, and induces anoikis resistance in MKN45 and KATOIII cells, which was also demonstrated in a xenograft model. Although the study cohort consisted of various GC stages, PC patients were also included, and the study results were validated on a PC xenograft model. 

The first study in this field focused on the dysregulation of microRNAs (miRNAs) came in 2018 by Schindler et al. [27], who showed miR-21, miR-186, miR-222, and miR-483-5p to be upregulated and miR-26b to be downregulated in PC compared to spontaneous bacterial peritonitis (SBP), a benign condition often mistaken with PC. MiRNA expression validation analysis confirmed higher expression levels of miR-21 and miR-186 in patients with PC, whereas miR-223 was significantly upregulated in patients with SBP. A simple proportion score between miR-21 and miR-223 allowed the authors to discriminate between the patients with PCA and those with SBP, with an area under the curve of 0.982 (95% confidence interval, 0.943–1.022). The undeniable value of this work comes from the fact that, as one of very few at the time, it provided an analysis of miRNA levels in accumulated peritoneal fluid, ascites, which accompanies intraperitoneal tumors, most commonly ovarian cancer [38,39]. The authors in this proof-of-concept study showed that the measurement of miRNAs in ascites is feasible and should be considered a viable option for future semi-invasive diagnostic purposes [27] since current diagnostic strategies such as CT and ultrasound lack the sensitivity to detect PC, causing the disease to be discovered much too late [3].

Heublein et al. [28] provided a very valuable comparison of miRNA expression in tumors with differing sites of metastatic invasion. They compared CRC tissue of tumors metastasizing to the liver and the peritoneal wall to non-metastatic CRC. The results showed that there is a specific miRNA expression profile typical for primary tumors spreading to the liver versus tumors that later form PC. Among the miRNAs identified as dysregulated in the exploratory phase, the qPCR validation showed miR-31 to be significantly downregulated the most in liver-directed tumors and less in PC-directed tumors than in non-metastasizing tumors. The team showed that miR-31 was downregulated by c-MET, a potent oncogene [28].

Similarly, a study by Aziret et al. [34] focused on miR-99b and miR-135b in PC and liver metastasis in patients with CRC and their association with KRAS and Akt expression. Liver metastases and eventual PC are quite frequent among patients with CRC [34,40,41]. Similarly to other articles discussed in this review, this publication also built on the previous association of miRNAs of interest with the development and progression of cancer [42,43,44,45,46]. The mutational status of NRAS and KRAS was determined and the levels of miR-135b and miR-99b were measured. While levels of miR-135b increased, miR-99b decreased in PC and liver metastases compared to primary tumor tissue; however, in general, the levels of both miRNAs were higher in primary tumor tissue than in normal tissue. The authors did not observe any correlation between Akt expression, KRAS mutations, and miRNA expression levels, which they considered to be an effect of the small cohort. However, they observed an association of miR-99b levels, KRAS mutations, and Akt with a higher risk of poor overall survival.

Di Agostino et al. [33] focused on peritoneal ovarian carcinomatosis and the molecular mechanism of HIPEC’s effect on cancer cells. HIPEC is currently the treatment of choice for patients with favorable health conditions for such a treatment. The study was specifically focused on miR-145, a well-known tumor suppressor miRNA. Dysregulation of miR-145 is associated with cancer progression and dissemination [47,48]. The authors first confirmed a significant decrease in miR-145 in metastatic tissue compared to adjacent normal tissue. Next, they investigated the miR-145 level dynamic after HIPEC treatment by sampling one metastatic module extracted during the cytoreductive surgery in specific time points and showing that miR-145 levels were restored to normal after HIPEC treatment. This was also successfully repeated using a cell culture model. They further identified four mRNA targets of miR-145, MYC, EGFR, MUC1, and OCT4, which were decreased after HIPEC, negatively correlating with the miR-145 level. Moreover, considering the “heated” component of HIPEC, the authors studied the relationship between miR-145 and heat-shock proteins, which are inevitably activated after the application of HIPEC, and found that HSF-1 is an important upstream modulator of miR-145.

## 4. Can We Leverage Ascites as a Source of Non-Coding RNAs?

As the topic of ascites is tightly associated with PC, there are already several other publications on the topic of ncRNA profiles in this body fluid. Interestingly, all of them analyzed miRNAs specifically originating from exosomes or, more broadly, from extracellular vesicles (EVs). On the importance of EVs, specifically exosomes in cancer, Li et al. [32] summarize that, “In particular, tumor derived EVs are prominent mediators for the intercellular communication between stromal cells and tumor cells in distant and local microenvironments, exerting a crucial role in both tumor primary growth and metastatic evolution [49]. Many of these functions are triggered by EV-encapsulated miRNAs [50].” Exosomes seem to be a running topic in the field of PC research, as these secreted particles are commonly present in ascites. Yun et al. [29] compared ascites of GC patients with ascites originating from liver cirrhosis. The study cohort was stratified into three independent subcohorts for the discovery, training, and validation phase of the study. Similarly to the results of Heublein et al. [28], the team proved that there was a specific exosomal miRNA profile in ascites of GCs versus liver cirrhosis ascites. The most significantly dysregulated miRNAs were miR-574-3p, miR-181b-5p, miR-4481, and miR-181d. Moreover, miR-181b-5p in combination with carcinoembryonic antigen (CEA), a common marker in gastrointestinal tumors, improved the CEA diagnostic performance. This study was the first to address exosomal miRNAs, let alone in malignant ascites in the context of PC. As the authors stated, “Ascites or peritoneal lavage is more suitable for evaluation of PC than primary tumor tissues or blood samples because of their closer proximity and direct contact with tumors within the peritoneum and omentum” [29].

The role of exosomes and exosomal miRNAs has also been studied by Hu et al. [30], who showed that exosomes isolated from malignant ascites can influence invasion via the EMT signaling pathway. Moreover, the study showed once again a specific miRNA profile in ascites of GC patients with peritoneal dissemination compared to controls and that this profile changed after intraperitoneal chemotherapy treatment. Using NGS, the study identified 160 differentially expressed miRNAs. Subsequent in silico miRNA target analysis highlighted miR-196, a pro-metastatic miRNA typically overexpressed in many types of cancer, including GI tumors. Although acquired in a small cohort of patients, the results are consistent with other evidence that exosomal cargo promotes peritoneal dissemination [51]. The study of Zhang et al. [35] presents similar results. The team consistently worked on chromobox protein 7 (CBX7) and showed that it is a potent tumor suppressor whose inhibition enables metastasis in OC [52]. In their subsequent study [35], they collected adipose conditioned media (ACM) from a human omental transplant and observed that after treating OC cell lines with ACM, the levels of CBX7 were downregulated and cells manifested increased invasiveness and migratory abilities. The team hypothesized that ACM-derived exosomes and their contents were responsible for the effect, and thus they carried out a characterization of EVs from ACM and analyzed exosomal miRNA content. During in silico target prediction of miRNAs targeting CBX7, they selected miR-421 as the miRNA of interest. The regulation of CBX7 expression by miR-421 was confirmed experimentally, and increased levels of miR-421 were measured in adipose-derived exosomes from OC patients, although the cohort consisted only of six patients. The authors concluded “that adipose microenvironment can alter the phenotype of OC cells” [35].

Promising results were brought by Lobos-Gonzalez et al. [31] using an in vivo PC model and in vitro breast cancer cell lines treated with antisense non-coding mitochondrial RNA (ASncmtRNA), which was earlier identified and described by the same team [53]. This very specific class of ncRNAs originates from mitochondrial 16S rRNA and comprises both sense and antisense ncRNA, with a potential role in cancer [53,54,55,56]. In a study by Lobos-Gonzalez et al. [31], exosomes derived from the BC cell line treated with ASncmtRNA inhibited tumorigenesis of recipient cells. ASncmtRNA knockdown using an antisense oligonucleotide caused massive death of tumor cells but not normal cells and strongly reduced metastasis in mice, suggesting that this new ASncmtRNA could serve as a new potential therapeutic target. 

Continuing with exosomal miRNAs, an interesting study by Li et al. [32] looked at the effect of gambogic acid, a promising new anticancer agent, on breast cancer cells and a PC mouse model. The mechanism of miR-21 and gambogic acid interplay, which was suggested earlier elsewhere [57], was investigated in the study of Li et al. The results show that gambogic acid decreased levels of tumor cell-originating miR-21 in EVs, thus affecting M2 polarization of macrophages, and limiting the invasiveness and liver metastases of CRC. However, as the authors note, they did not investigate the downstream targets of miR-21 focused only on the effect on macrophages, and worked with only one cell line, outlining the space for further research.

## 5. Research Challenges and Future Directions

PC, as indeed a challenging fatal condition, needs much more scientific attention, although recent advancements in medical research have shed some light on the nature of this disease. The focus on ncRNAs adds a novel dimension to PC exploration, given their pivotal role in cellular regulation. 

The design of the reviewed studies is diverse both in methodological approach and samples used, although exosomal miRNAs from ascites are the preferred source of material, considering the relative proximity to the tumor tissue. However, independent validation on larger cohorts is needed. Only three studies attempted high-throughput profiling [27,28,29] using microarray or PCR array in both tissue and ascites, while other studies focused on one or two selected ncRNAs potentially involved in the development of PC based on previous research on peritoneal metastases, but not in PC specifically. Although very similar in nature, PC presents a specific state that could have a distinct ncRNA profile compared to less disseminated disease. The application of modern high-throughput profiling methods such as next-generation sequencing and its variations, for instance, single-cell sequencing, which has already been employed in ascitic fluid samples [58], could help identify novel ncRNAs specific for this condition.

Moreover, venues of liquid biopsy using ascites should be explored deeper. Current diagnostic techniques combined with the asymptomatic nature of PC are adding to the late diagnosis and, by extension, to limited, if any, therapeutic options for patients. After all, a hunt for earlier discovery options and tools is a pressing need in many other types of cancer as well [3]. Specifically in the case of PC, and by extension intraperitoneal tumors, ascites is promising source of biomarkers potentially feasible in the diagnosis and prognosis assessment [3,59,60], as is also evident from the results summarized in this review showing that ncRNA profiles in ascites could help distinguish PC from non-malignant conditions [28,29] similarly to other tumors of the intraperitoneal cavity and non-malignant counterparts [39]. On the other hand, blood-borne biomarkers also present an option for non-invasive discovery of PC [3] or the prediction of its development, especially in cases when ascites is not present. In recent decades, miRNAs have been shown to be potential biomarkers with great sensitivity and specificity, correctly identifying several cancers of unknown origin with considerable accuracy [61,62]. Most studies discussed in this review analyzed only small numbers of ascites samples, with some exceptions that processed larger cohorts [26,29,34]. There is currently no study on PC employing serum or plasma samples. Other studies reviewed in this article relied on small, biased cohorts; therefore, independent validation is needed before any conclusion can be drawn, as currently, no overlap in identified miRNAs is evident. Further, comparing lower-stage and advanced disease with PC could help identify prognostic ncRNAs typically dysregulated in patients with a higher risk of developing PC. More research is necessary in this underexplored area. 

## 6. Conclusions

In conclusion, this review aimed at the consolidation of current, limited knowledge on the role of non-coding RNAs in the development of PCs. Independent validation in bigger cohorts is needed, along with the application of high-throughput methods. Ascites and serum should be explored as liquid biopsy sources of biomarkers, including ncRNA, in diagnosis, prognosis, and therapeutic outcome prediction. Further research in these areas could also improve the prognosis and survival of patients with PC.

## Figures and Tables

**Table 1:** Articles focused on the dysregulation of ncRNAs in peritoneal carcinomatosis, and overview of studies identified within the literature search for this review. Abbreviations: BC = breast cancer, CL = cell lines, CRC = colorectal cancer, FFPE = formalin-fixed paraffin-embedded, GA = gambogic acid, GC = gastric cancer, H&E = hematoxylin/eosin staining, ChiP = chromatin immunoprecipitation, IHC = immunohistochemistry, LCi = liver cirrhosis, LM = liver metastases, M0 = advanced CRC cases without metastases, NT = non-tumor, OC = ovarian cancer, OS = overall survival, PC = peritoneal carcinomatosis, PH = portal hypertension, SBP = spontaneous bacterial peritonitis, T = tumor, TEM = transmission electron microscope, TLDA = TaqMan low-density array, TS = tumor suppressor, WB = Western blot.

Disease [Reference]	Significant ncRNAs	No. of Samples/No. of Patients, Material	Methodology	Findings
**GC [26]**	MALAT1, HOTAIR	300/150, paired tumor and non-tumor tissue, GC cell lines, mice	qPCR, functional in vitro functional tests, in vivo tests	Both lncRNAs↑ in GC patients with peritoneal metastasis; HOTAIR↑—poor outcome, promotes metastases, tumorigenicity, invasiveness.
**PC vs. SBP vs. PH [27]**	miR-21, miR-223, miR-186, miR-26b	45/45 ascites, PC (n = 15), SBP (n = 15), PH (n = 15)	TLDA, qPCR validation	↑miR-21, miR-186, miR-222, miR-482-5p, ↓miR-26b; miR-21 and miR-186↑ overall in PC vs. PH.
**CRC LM vs. PCa vs. M0 [28]**	miR-31-5p	10/10 CRC LM, 10/10 CRC PCa, 3/3 CRC M0, FFPE	TLDA, qPCR validation, IHC, WB	miR-31-5p↑ in PC-directed CRC, repressing c-MET, role in invasivity, predicts metastatic spread site.
**GC [29]**	miR-574-3p, miR-181b-5p, miR-4481, miR-181d	165/165 ascites samples (LCi-ascites n = 73, GC-ascites n = 92)	Microarray, qPCR, immunoassay	Combination of CEA and exo-miR-181b-5ppotential diagnostic biomarker of non-malignant vs. GC-ascites.
**GC [30]**	miRNA panel	16/8 GC ascites, 3/3 Lci ascites, AGS cell line	TEM, WB, RNAseq, invasion assay, in vivo model	Ascites-derived exosomes promote GC invasion by inducing EMT signaling, panel dysregulated in GC pre- vs. GC post-treatment, GC vs. LCi.
**BC [31]**	ASncmtRNA	BC cell lines (MDA-MB-231, ZR-75, MCF-7)	TEM, nanoparticle tracking analysis, WB, qPCR	Exosomes released upon ASncmtRNA knockdown reduce tumorigenic properties of BC cells.
**CRC [32]**	miR-21	CRC cell lines HT-29, SW480, HCT116; normal (HCO), mice	qRT-PCR, H&E, in vitro and in vivo tests, ELISA	GA interferes with M2 polarization of macrophages by suppressing tumor EV-miR-21, thus weakening metastasis and invasion.
**OC [33]**	miR-145	20/10 FFPE (T + NT), time-point sampling of the metastatic nodule, OVCAR-3 and ES2	IHC, qPCR, pyroseq, in vitro tests, WB, ChIP	miR-145-5p and its targets (c-MYC, EGFR, MUC1, and OCT4) impaired in peritoneal metastases, HIPEC restored the TS activity of miR-145-5p, HSF-1 is the upstream regulator.
**CRC [34]**	miR-99b, miR-135b	137/74, FFPE, PC CRC (n = 46), LM CRC (n = 28)	Mutation status detection, IHC, RT-PCR	miR-99b↓, miR-135b↑ in PC and LVM vs. primary tumor, Akt, KRAS and miR-99b mutations are potential risk factors for poor OS.
**OC [35]**	miR-421	6/6, omentum, OVCA432, OVCAR3	EV characterization, ExoView profiling, in vitro tests, WB, qPCR	miR-421/CBX7 leads to↓CBX7, a major part of the histone-modifying PRC1.

## Data Availability

No new data were created or analyzed in this study. Data sharing is not applicable to this article.
